# A Pregnant Patient Presenting With Unilateral Sacroiliitis Following Dengue Hemorrhagic Fever: A Case Report

**DOI:** 10.7759/cureus.14946

**Published:** 2021-05-10

**Authors:** HKDK Prabasara, Mayurathan Pakkiyaretnam, Maheswaran Umakanth

**Affiliations:** 1 Internal Medicine, Teaching Hospital, Batticaloa, LKA; 2 Clinical Medicine, Teaching Hospital, Batticaloa, LKA

**Keywords:** unilateral sacroiliitis, dengue virus infection, complications of dengue fever, etiologies for sacroiliitis, treatment for dengue sacroiliitis

## Abstract

Dengue virus infection is an arthropod-born infection with high global prevalence. A spectrum of clinical syndromes and complications were recognized following dengue fever that can range from undifferentiated fever to dengue shock syndrome. Neurological complications following dengue fever can extend to various sequelae, including transverse myelitis. We report a 20-year-old pregnant woman with a recent diagnosis of dengue hemorrhagic fever (DHF) at the period of amenorrhea (POA) of 28 weeks, presenting with left-sided severe buttock pain. Following extensive investigations, we found left-sided sacroiliitis to be the cause of the buttock pain. She completely recovered with appropriate management.

## Introduction

Dengue virus is an arthropod-born infection with high burden on the community in Sri Lanka, indicated by 31,017 reported cases for the year of 2020 despite biological and chemical vector control measures [1]. Aedes aegypti and Aedes albopictus are two type of vectors contributing to the spread of dengue fever [2]. Dengue fever presentations can vary from undifferentiated fever to dengue hemorrhagic fever or dengue shock syndrome. Myocarditis, encephalitis, cholecystitis, hepatitis, transverse myelitis, and Guillain-Barre syndrome are other reported complications of dengue fever [3-5]. Sacroiliitis is a recognized unusual manifestation of dengue fever commonly reported after 10 days of dengue viral infection [2]. Dengue viral illness is associated with arthralgia and myalgia although it is not known to cause arthritis. Viral arthritis is recognized following rubella, parvo, hepatitis B, hepatitis C and chikungunya viruses [2]. Our patient is a 20-year-old pregnant mother with a recent history of dengue hemorrhagic fever (DHF) who presented with a complaint of severe left-sided buttock pain. She was extensively investigated and found to have left-sided sacroiliitis. We excluded possible etiologies for sacroiliitis and finally concluded this was due to dengue fever.

## Case presentation

A 20-year-old pregnant mother with a period of amenorrhea (POA) of 28 weeks was admitted to our tertiary care centre due to left-sided severe buttock pain, five days prior to admission. The pain was very severe and she could not even walk due to the pain. She has taken paracetamol as over-the counter-medication and had no satisfactory response. She had a recent history of DHF, which we treated with appropriate fluid management and supportive care, and was discharged eight days before after complete recovery. Initially, she was admitted to a local hospital due to buttock pain for which paracetamol was given as a painkiller, with minimal response. Due to the short duration of her condition and unavailability of facilities, no further investigations were conducted during the local hospital admission. Following her discharge, she experienced the same kind of pain again, after which she was admitted to our tertiary care centre. She did not have other small or large joint pain, swelling and there were no features to suggest enthesitis. She did not have fever, dysuria, vaginal discharge, or skin eruptions. There was no recent history of sore throat or diarrheal illnesses. She also had no past history of joint pain, photo-sensitive skin rashes, oral ulcers, or alopecia. There was no past history of altered bowel habits or contact history of tuberculosis.

**Figure 1:**
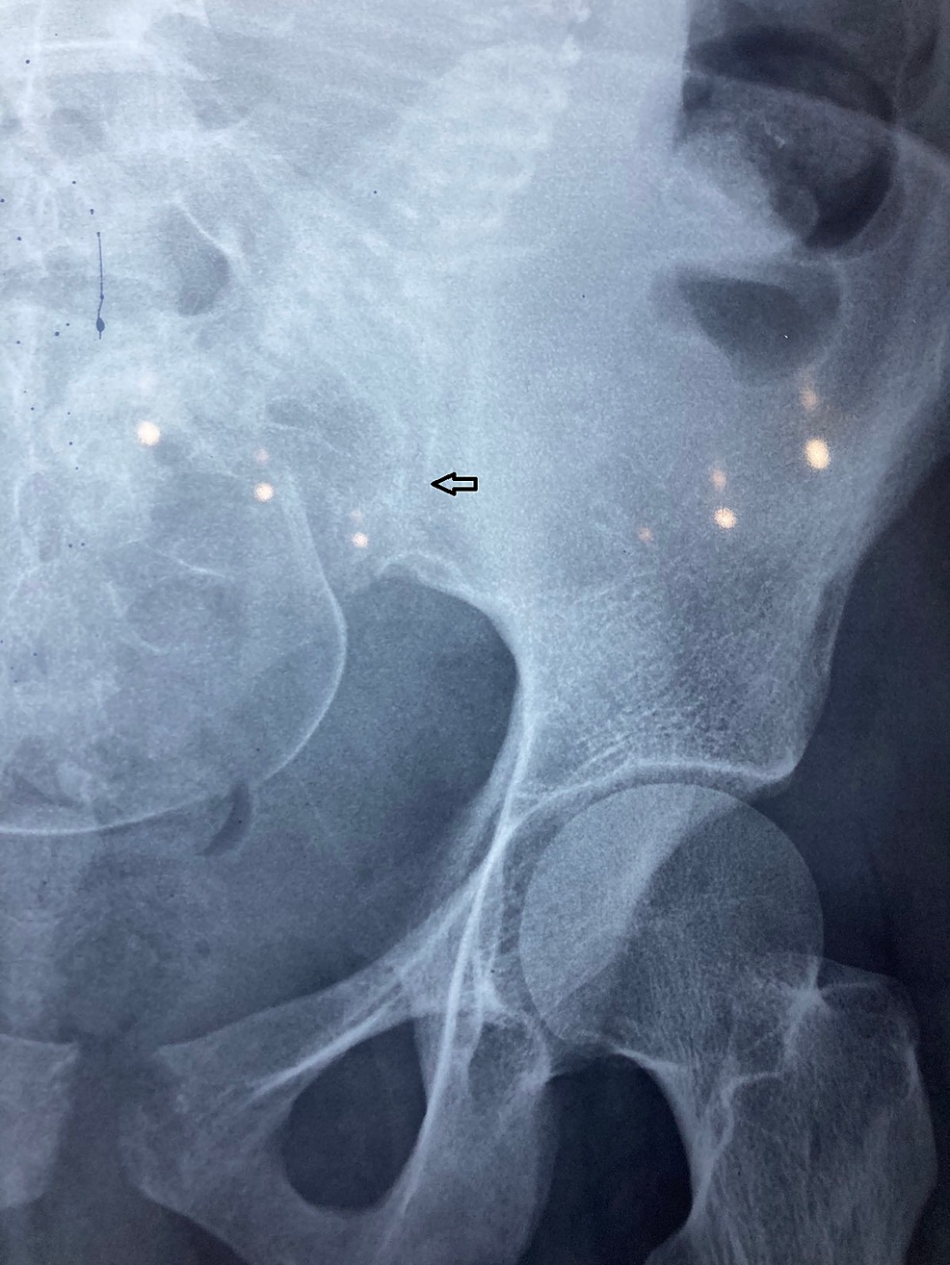
Left-sided sacroiliac view The arrow indicates the blurring of the left sacroiliac joint, indicating left-sided sacroiliitis.

**Figure 2:**
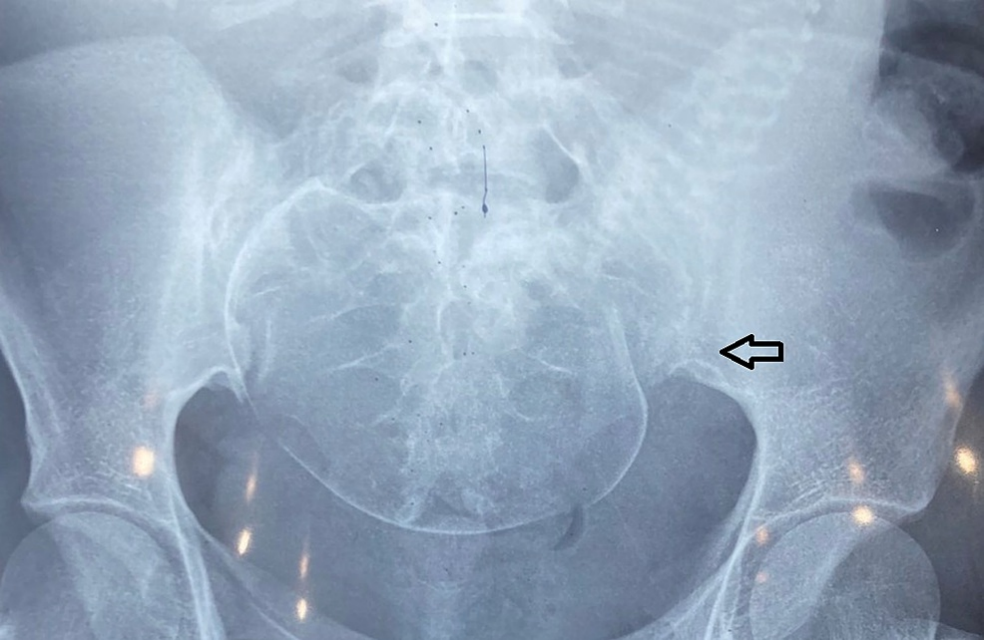
X-ray bilateral sacroiliac view showing left-sided sacroiliitis with fetus in-situ The arrow shows left-sided sacroiliac joints (affected side) contrasted with normal right-sided sacroiliac joint.

On general examination, she was not pale or icteric. There was no red eye, malar rash, oral ulcers, or any peripheral stigmata to suggest ongoing vasculitides. Respiratory system examination was normal and did not reveal any features to suggest lung fibrosis. Cardiovascular system examination revealed mild tachycardia (110 beats per minute) and normal blood pressure. There was no evidence of aortic regurgitation or mitral valve prolapsed. Abdomen was non-tender and had no hepato-splenomegaly. The nervous system and peripheral joint examination were normal. X-ray of her sacroiliac joint revealed features of left-sided sacroiliitis which was evidenced by blurring of joint margins with minimal sclerosis of the left sacroiliac joint (see X-rays in Figures 1-2). Full blood count (FBC) revealed white blood cells 14000/µL of which 77% were neutrophils. Hemoglobin concentration was 10 g/dl and platelets 553000/µL. Erythrocyte sedimentation rate (ESR) was 103mm/1st hour and CRP was 9 mg/dl. Dengue IgM and IgG both were positive. Urine full report, liver function tests, renal function tests, and uric acid were within normal range. Blood and urine culture both were sterile. Tuberculin skin test was negative. Antinuclear antibody (ANA) and rheumatoid factor were negative. Retroviral, Hepatitis B and C screenings were negative. Human leukocyte antigen (HLA B27) was negative. Thyroid function tests and iron studies all were within normal limits. We managed her with regular paracetamol and oral tramadol appropriately to alleviate her pain and she gradually improved over the next week. We reviewed her after two weeks with FBC and ESR. At her two-week review, the white blood cell count was down to 12200/µL and platelet count was 365000/µL. Her ESR report was 43 mm/1st hour. After two weeks, significant clinical and biochemical improvement was observed following appropriate symptomatic management.

## Discussion

Dengue fever is an undifferentiated viral illness that can progress to life-threatening complications like dengue shock syndrome. Although arthralgia is one of the commonly encountered symptoms related to dengue, arthritis is rare and only a few case reports exist as evidence [2]. Our patient is a 20-year-old pregnant mother who was recently treated for dengue hemorrhagic fever in our tertiary care centre. Following the third day of hospital discharge, she developed left-sided severe buttock pain. The pain started 11 days following the onset of dengue fever. Available case reports reveal the usual time lag to develop sacroiliitis as 10 days from the onset of fever [2]. The absence of fever or constitutional symptoms at the time of presentation as well as normal CRP and negative cultures were the clinical evidence against infective etiology. Some bacterial infections are known to pre-direct its joint involvement towards the sacroiliac joint. Brucellosis, tuberculosis, staphylococci, and pseudomonas aeruginosa are reported pathogens with sacroiliac joint predilection [2-6]. Considering the very low prevalence of brucellosis in Sri Lanka brucellosis serology was not conducted for our patient. Our patient had no past history of diarrhea or alteration of bowel habits, history of joint diseases or enthesitis, uveitis, conjunctivitis, or urethritis. The absence of former symptoms made seronegative spondyloarthropathies unlikely as the etiology which was further confirmed by negative HLA B27. There are several postulated mechanisms believed to cause arthritis secondary to viral infections. Direct invasion of the synovium by the virus and immune complex deposition are among the most likely hypotheses [2]. Considering the above two hypotheses, there is a strong possibility of dengue virus caused arthritis, which was the etiology in our patient and as well as in several case reports that were published recently [2]. Viral arthritis is usually self-limiting and unlikely to last more than several weeks. No specific treatments recommended due to the self-limiting nature of the disease, as was seen in our patient.

## Conclusions

Unilateral sacroiliitis is a sequale of dengue fever which is usually self limiting and if necessary need supportive treatments with appropriate analgesics. Any patients presents with significant back pain or buttock pain following dengue fever, sacroiliitis should be considered as a possible differential diagnosis and appropriate investigations are recommended.
